# Supplementary material for: An alignment-free strategy for circulating tumor DNA detection and tumor fraction estimation from whole-genome sequencing data
Source: PLoS Comput Biol. 2026 Aug 10;22(8):e1013356. doi: 10.1371/journal.pcbi.1013356 (PMC13485129; doi:10.1371/journal.pcbi.1013356)
Supplement: Supplementary file 1 [file pcbi.1013356.s019.pdf]

Summary:

ctDNA is a promising new direction for monitoring post-operative cancer patients. However, for ctDNA to be effective as a monitoring biomarker, ctDNA must be demonstrated to be a highly sensitive indicator. Although current targeted approaches have better sensitivity to detect a specific mutation, recent literature also indicate that WGS data allows us to characterize tumor-specific mutations to a wider range of variants (SNVs, CNAs, etc). Existing work leveraging WGS data for ctDNA detection requires alignment to a reference before performing variant calling. Both of these steps have several confounding factors which have been demonstrated to strongly influence the tumor fraction estimation.

This paper presents ctDNAmer, which avoids reference alignment by identifying k-mers unique to the tumor sample by using primary tumor and germline WGS data, with some modeling of the noise affecting the circulating tumor fraction estimate. The authors model unique tumor (UT) k-mers with a three-component mixture model corresponding to the three ways of generating unique tumor k-mers. The parameters corresponding to the sample-specific noise and missed germline variant are empirically estimated and fixed for subsequent analysis.

The topic seems to be a timely and impactful one. However, as the authors use existing KMC3 tools for the k-mer manipulations, the main contribution seems to be the novelty of demonstrating that reference-free ctDNA detection is possible and the mixture model of k-mer classification. I have some concerns on the clarity of the method presented, as well as the extent to which this contribution has been probed.

Major concerns:

- Given that the filtering of the UT k-mer sets appears to be the first step in ctDNAmer, I am concerned about the justifications provided for each step. Although there is a count-filtering heuristic and experiment in 3.2 to determine the optimal minimal UT set size, the explanation for the lower and upper cutoffs seems arbitrary. It's unclear how much these affect the downstream tumor fraction inferred.
- Overall, the inputs and outputs to ctDNAmer are confusing. In Section 5, it seems that unmatched cfDNA samples are used, but compared to matched cfDNA data. Does this mean that unmatched and matched data are necessary? Are there rare variants that appear consistently over time when given longitudinal data? Could these be getting filtered out? How do the authors handle changing tumor heterogeneity?
- Are the noise distributions estimated per sample? Is μ set to 0.01 because that was the empirical value? What is the effect of leveraging these priors?
- Since both the initials for both the noise and overdispersion parameters seem very low, it would seem that perhaps a maximum likelihood approach is more computationally efficient (especially since the authors later cite computational efficiency as an advantage).
- Although different k-mer lengths were tried, it is unclear that the variants identified across the different k-mer lengths are the same. Given that heterogeneity in variants

should be one of the advantages of ctDNAmer, it seems likely that a profile of different k-mer lengths could better characterize a patient.

- I believe the authors claim an improvement in computational efficiency since ctDNAmer does not rely on alignment to a reference. It would be good to provide a runtime and memory efficiency comparison to characterize the advantages and disadvantages of such a k-mer based approach.
- In Figure 2f, it seems that ctDNAmer actually does differentiate across the samples characterized as negative, whereas clonal SNVs approach does not. This would seem to be a disadvantage of ctDNAmer. Can the authors add a discussion reconciling this result with the higher AUC for ctDNAmer?
- The authors could consider discussing the role of minimizers in this application.
- One of the key advantages to ctDNAmer is the ability to identify different variant types and thus improve detection sensitivity. Could the authors compare the variants identified by ctDNAmer with the alignment + variant calling approaches?
- Could the k-mer length analysis be extended to evaluate whether there is a representative sample of SNVs, complex variants or structural variants at different lengths?
- Could the authors provide a more intuitive explanation for why approaches leveraging additional reference data do not produce better tumor fraction estimates than ctDNAmer?
- A set of simulations would help the reader understand the role of modeling the two sources of noise.
- I have checked the github and read some of the additional links, but did not try running the method. I would suggest to the authors to upload a small test dataset to make it easy for the user to try running. Also, some of the links on https://github.com/BesenbacherLab/ctDNAmer/tree/main/config seem to be broken.
- (minor) cfDNA definition
