# Supplementary material for: An alignment-free strategy for circulating tumor DNA detection and tumor fraction estimation from whole-genome sequencing data
Source: PLoS Comput Biol. 2026 Aug 10;22(8):e1013356. doi: 10.1371/journal.pcbi.1013356 (PMC13485129; doi:10.1371/journal.pcbi.1013356)
Supplement: Supplementary file 2 [file pcbi.1013356.s021.pdf]

Point-by-point responses to the reviewers

Below, reviewers' comments and questions are shown in black, and our responses in blue.

Reviewer #1

In this manuscript, Oroperv and colleagues propose a reference free, “tumor informed” cfDNA approach based on k mer analysis across three matched samples per patient, tumor, germline, and plasma cfDNA; each sequenced at ~30× whole genome depth (on the order of hundreds of millions of fragments per sample). The model is then applied to a cohort of stage III colorectal cancer patients (n=90) with three year follow up.
From a computational/bioinformatics standpoint, the technique is novel and could have utility beyond the specific use case studied. However, as presented, the performance and clinical applicability are unclear and underwhelming. The strategy effectively requires ~90× WGS per patient (3×30×), yet the detection limit appears to lie between ~0.1% and 1% (Figure 2F), which substantially limits clinical utility and is not competitive in the MRD setting. By comparison, simple targeted capture methods focusing on recurrent SNVs can reach limits around ~0.01% at roughly an order of magnitude lower cost than ~90× WGS. Moreover, for standard ~30× WGS, alignment is performed in routine workflows; thus, the “reference free” paradigm does not realistically prevent mapping. That said, I would be interested to see whether k mer–based signals provide orthogonal information that synergizes with post alignment features (e.g., variants, copy number, indels, fragment length).
I encourage the authors to consider reframing the contribution toward contexts such as novel sequence identification or motif discovery, rather than MRD detection or circulating tumor fraction estimation. Hence, I recommend major revision.

We appreciate these comments and the recognition of the novelty of the proposed method. We agree with the reviewer’s observation that the k-mer-based technique has potential applications beyond the presented use case of postoperative monitoring. At the same time, we acknowledge that the tested colorectal cancer cohort sequenced at ~90x WGS coverage across three samples per patient may not currently be the most cost-effective strategy for MRD detection. Nevertheless, we believe there is considerable value in investigating novel analytical strategies for matched WGS data and hope the reviewer agrees that this work remains both valuable and publishable, especially with the inclusion of additional analyses incorporated during revision, which further emphasize the method's potential and practical utility.

Importantly, we agree that applying a reference-free method does not preclude or replace alignment-based analyses. An ensemble approach combining these two orthogonal methodologies could further enhance recurrence detection performance. This rationale also underpins our view that WGS-based ctDNA detection holds unique advantages over targeted approaches.

Major Comments

1. The k mer analysis is highly computationally intensive, requiring analysis of billions of reads. Without alignment, it is also difficult to confirm the biological validity of findings—e.g., whether observed signals correspond to mutations relevant to the cancer type of interest.

We agree that there are considerable computational requirements for carrying out the proposed k-mer-based analysis on WGS data. Nevertheless, we find that similar or even more intensive requirements are needed when analyzing the same data using read alignment and variant calling.

It is true that without read alignment, the biological validity of individual UT k-mers cannot be confirmed. Nevertheless, we believe that the concordance we observe between our k-mer-based ctDNA detection results and clinical data, an alignment-based ctDNA detection approach, and targeted ctDNA detection methods indirectly show that the unique tumor k-mers capture true biological signals. Also, we expect the tumor-specific unique tumor k-mers to capture somatic mutations present in the given individual's cancer cells rather than driver mutations relevant to a specific cancer type.

To further confirm the biological validity of the proposed method, we aligned UT k-mers to the human reference genome and analysed the alignment results by comparing aligned UT k-mers overlap with SNVs, indels and structural variants called with Mutect2 and Delly tools. The analysis results are described in Results section 3 subsection and shown in Supplementary Figure 5, and demonstrate that UT k-mers capture true tumor variants of different types.

2. Please comment on the impact of PCR induced duplications on the k mer counts and downstream results. How are duplicates detected/handled, and how sensitive are results to duplication rates?

Indeed, PCR-induced duplications, as well as k-mers observed twice due to the overlapping of paired-end cfDNA sequencing reads, will increase the cfDNA k-mer counts, and in the reference-free setting, duplicate removal cannot be performed, as this is usually done as a post-alignment filtering step. However, we expect PCR duplicates to affect cfDNA sequencing reads originating from tumor cells as well as reads originating from healthy cells at a similar rate, and the model accounts for the mean cfDNA count when modeling cfDNA k-mer counts. Therefore, we assume that the estimated circulating tumor fractions are scaled correctly regardless of the PCR duplicates. This is confirmed indirectly by Figure 2F, which shows that the estimated circulating tumor fractions are on the same scale and strongly correlate with the TF estimates from the analysis of aligned data, where duplicates were removed after read alignment. Therefore, we expect the PCR duplicates not to affect the results on a large scale.

3. The selected k values (31, 41, 51, 61) appear primarily empirical. While k=51 performed best in FRFR samples and k=31 in FFPE samples, the rationale for limiting the search to this range is not fully clear. Why were multi k strategies not explored? Since the optimal k seems to differ by sample type, please discuss whether broader sensitivity analyses or adaptive k mer selection might improve robustness across sequencing contexts.

We agree that a broader sensitivity analysis could provide a more comprehensive overview of the robustness and limitations of the proposed method. We also agree that adaptive k-mer selection might improve the method's robustness across different sequencing settings, as exemplified by the difference in FRFR and FFPE cohorts. However, we find that these analyses are out of scope for this proof-of-concept manuscript and could be explored in future studies, along with the robustness of the method across cancer types, tumor purity levels, and sample coverage. We have added a note about this to the Discussion section.

We have added the k-mer length of 21 to the k-mer length comparison analysis (k-mer length of 11 is not feasible, as there are no UT k-mers as short). K-mers longer than 61 bp could be explored, but will result

in longer running times and are not expected to improve ctDNA detection results further due to trends seen across k-mer length comparison analysis.

Multi k strategies were not explored, as we assume results across k-mer lengths to correlate strongly. Applying a multi-k ensemble approach, either by averaging predictions across different k-mer lengths or by using another summary statistic, might improve consistency, but we do not expect a large improvement in ctDNA detection sensitivity. This is supported by the correlation of TF estimates across k-mer lengths. We have added the TF estimates correlations across k-mer lengths as a Supplementary Table 1 for FRFR and FFPE.

4. On page 21, the manuscript suggests that smaller k mers yield better results, which contrasts with earlier choices and highlights the framework's sensitivity to k. This raises robustness concerns that should be addressed explicitly.

While we do not find a note of this on page 21, the reviewer's comment is correct and smaller k (k=31) did yield better results for the FFPE cohort. And indeed, this contrasts with FRFR results, where the best result was achieved with k=51, indicating that the optimal k may vary with sample quality and perhaps other covariates. We understand the concern regarding the method's robustness, but given that FFPE samples with elevated noise rates can also affect method performance, including alignment-based approaches [1], we hope the results still provide substantial evidence that the k-mer-based method is sufficiently trustworthy. However, as mentioned in the reply to question 3, we agree that further analyses to explore the method's robustness could be carried out. To further explore the robustness of the method in different settings, we have added an analysis of k-mer length of 21 and a LOD analysis with multiple replicates.

5. Consider mapping reads carrying "germline" k mers back to the reference and testing for enrichment of known population SNPs (e.g., from gnomAD) within a statistical framework to substantiate the interpretation of these signals.

We agree that the proposed analysis of "germline" k-mers would shed light on the content of the k-mers subtracted from the tumor samples and we do expect a subset of the "germline" k-mers to capture population SNPs. That being said, the "germline" k-mers can also capture germline variants that are rare in the population or specific to the individual and therefore not present in gnomAD. Instead of an analysis of aligned "germline" k-mers, we hope that the analysis of aligned UT k-mers provides support for the interpretation of the captured signal and provides proof of biological validity (see UT k-mers mapping results in result section 3 and Supplementary Figure 5).

6. When reporting sensitivity and specificity, please also provide sensitivity at clinically relevant fixed specificity thresholds (e.g., 95% or 98%), which facilitates comparison to established assays.

We have added sensitivity values at a fixed specificity threshold of 95% to results section 3 (Tumor fraction estimation for postoperative monitoring of stage III colorectal cancer patients), and we show the corresponding confusion matrices for the detection results in Supplementary Figure 3.

7. The manuscript lacks an LOD analysis, which is standard for evaluating detection frameworks. This could be addressed via in silico spike in (mixture) experiments across a range of tumor fractions.

We have added a LOD analysis based on a set of in silico admixture samples to the manuscript. The analysis results are introduced in Results section 3 and shown in Supplementary Figure 6.

Reviewer #2

ctDNA is a promising new direction for monitoring post-operative cancer patients. However, for ctDNA to be effective as a monitoring biomarker, ctDNA must be demonstrated to be a highly sensitive indicator. Although current targeted approaches have better sensitivity to detect a specific mutation, recent literature also indicate that WGS data allows us to characterize tumor-specific mutations to a wider range of variants (SNVs, CNAs, etc). Existing work leveraging WGS data for ctDNA detection requires alignment to a reference before performing variant calling. Both of these steps have several confounding factors which have been demonstrated to strongly influence the tumor fraction estimation.

This paper presents ctDNAmer, which avoids reference alignment by identifying k-mers unique to the tumor sample by using primary tumor and germline WGS data, with some modeling of the noise affecting the circulating tumor fraction estimate. The authors model unique tumor (UT) k-mers with a three-component mixture model corresponding to the three ways of generating unique tumor k-mers. The parameters corresponding to the sample-specific noise and missed germline variant are empirically estimated and fixed for subsequent analysis.

The topic seems to be a timely and impactful one. However, as the authors use existing KMC3 tools for the k-mer manipulations, the main contribution seems to be the novelty of demonstrating that reference-free ctDNA detection is possible and the mixture model of k-mer classification. I have some concerns on the clarity of the method presented, as well as the extent to which this contribution has been probed.

We greatly appreciate this feedback and the recognition of the work as both timely and high-impact. We confirm that the primary objective of the manuscript was to provide a proof-of-concept for reference-free ctDNA detection, and we agree that the central novel contribution lies in the probabilistic three-component model used to categorize unique tumor k-mers and estimate circulating tumor fractions. We acknowledge the need for additional clarification of the presented method and for a more detailed presentation of the modeling results to validate the approach. We have revised the manuscript text and figures to address these points.

Major concerns:

1. Given that the filtering of the UT k-mer sets appears to be the first step in ctDNAmer, I am concerned about the justifications provided for each step. Although there is a count-filtering heuristic and experiment in 3.2 to determine the optimal minimal UT set size, the explanation for the lower and upper cutoffs seems arbitrary. It’s unclear how much these affect the downstream tumor fraction inferred.

The UT k-mer sets filtering strategy was created ad hoc, and finalized based on empirical results. We agree that the final patient-specific lower and upper cutoffs for k-mers' tumor counts, used for UT set filtering, are not intuitive, and based on the presented results, it is difficult to objectively evaluate how changing these cutoffs affects the TF estimation results. Nevertheless, we emphasize that the cutoffs are set so that k-mers that are kept in the UT set represent the peak of the tumor signal (mean tumor k-mer count) and the experiment from 3.2 further confirms that the TF estimation results are consistent regardless of the cutoff, down to a certain size of the UT set, where reliable estimation can still be performed (Supplementary Figure 11 shows flat, steady TF estimates across patients, regardless of increasing the lower cutoff).

To be more transparent about the final patient-specific filters applied, we have included a Supplementary Table 3 in the revised version that shows the final applied count cutoffs along with the mean tumor count and final size of the UT set for each patient in the colorectal cancer cohort.

2. Overall, the inputs and outputs to ctDNAmer are confusing. In Section 5, it seems that unmatched cfDNA samples are used, but compared to matched cfDNA data. Does this mean that unmatched and matched data are necessary? Are there rare variants that appear consistently over time when given longitudinal data? Could these be getting filtered out? How do the authors handle changing tumor heterogeneity?

Method section 5 introduces the sample-specific background noise estimation procedure. The reviewer has the correct impression that a set of unmatched plasma samples is used to create the data for estimating the noise rate empirically (by intersecting the UT set and the unmatched cfDNA k-mer sets one by one). Unmatched samples (cfDNA from other individuals, either patients or healthy donors) are necessary for noise rate estimation, as they provide k-mer sets in which we expect to see no k-mers specific to the patient's tumor or germline. Therefore, we assume that all k-mers we observe are present due to technical noise. Matched samples (cfDNA samples from the given patient) are not needed for noise rate estimation and are only used for the TF estimation.

If the reviewer refers to Supplementary Figure 1, where the average number of UT k-mers observed in unmatched cfDNA is compared to the average number of UT k-mers observed in matched cfDNA, and stratified by the k-mer count in the two cfDNA sample groups, this analysis was performed as a distinct exploratory analysis to show that the noise rate estimated from unmatched samples is a good proxy for the noise rate in the matched samples. The noise rates estimated from unmatched samples need to represent the noise rate in matched samples, to ensure that the estimated noise rate parameters do not bias the TF estimation. The analysis presented in Supplementary Figure 1 is not part of the noise rate estimation or TF estimation, but shown as proof that our expectation of unmatched samples providing a fitting set of k-mers for noise rate estimation is met.

Rare variants can appear several times across the longitudinal samples; however, it is less likely to observe rare variants across longitudinal samples compared to more frequent (clonal) tumor variants. We do not expect to filter out these variants with empirical noise rate estimation. Some k-mers representing rare variants are likely to be filtered out during UT set filtering by excluding k-mers with low tumor counts; however, we accept this trade-off to remove more k-mers representing background noise.

Temporal tumor heterogeneity is currently not accounted for in the analysis, as we only rely on k-mers observed in the primary tumor. If, during tumor evolution, the tumor clonal architecture and the contributions of different subclones change considerably, this could possibly reduce sensitivity for ctDNA

detection. In the future, tumor temporal heterogeneity could be accounted for by including k-mers observed in the cfDNA sets and not present in the primary tumor or germline sets into the analysis. But it is by no means obvious that such would lead to improved detection, and we decided that experimenting with it was out of scope for the current proof-of-concept manuscript and should rather be explored in future publications.

3. Are the noise distributions estimated per sample? Is μ set to 0.01 because that was the empirical value? What is the effect of leveraging these priors?

The noise distributions are estimated per patient (specifically, the patient-specific UT set). As we apply the same UT set without modifications across longitudinal monitoring, we do not expect the number of noise k-mers in the UT set to change, and the same background noise distribution can be used for the noise component in the TF estimation mixture model. The initial value of 0.01 is not empirically derived from the noise rate data, but set as a value close to the mean of the prior Beta distribution. Alternatively, we could choose the initial values following the Empirical Bayes methodology. However, the initial value at which the Markov Chains start should not affect the posterior samples if sufficient burn-in is performed and all chains converge to the posterior distribution, as we have confirmed based on model diagnostics.

The effect of the priors for the empirical distribution estimation is expected to be minute, as the effective sample sizes of the priors (30+1=31 for the $\mu_{N, emp}$ beta prior, and 1 for the $\phi_{N, emp}$ exponential prior, if considering it as a Gamma(1,1) distribution, it correspond to) are small compared to the 20,000+ UT k-mers we are modeling that provide the signal from the data. We, however, did not use completely uniform priors, and thus rely on prior information in rare cases where no noise k-mers are observed and the model needs prior information to converge.

4. Since both the initials for both the noise and overdispersion parameters seem very low, it would seem that perhaps a maximum likelihood approach is more computationally efficient (especially since the authors later cite computational efficiency as an advantage).

We clarify that the initial values of 0.01 for $\mu_{N, emp}$ and 0.1 for $\phi_{N, emp}$ are only the starting values for the Markov Chains, and in case of successful model convergence (which we have confirmed based on model diagnostic measures), should not affect the posterior samples. A maximum likelihood approach could also be applied here for model fitting, however, we prefer to keep a unified modeling approach across the two models (noise rate and TF estimation), as computational efficiency does not need to be improved. The noise rate estimation takes on average 7.1 minutes.

5. Although different k-mer lengths were tried, it is unclear that the variants identified across the different k-mer lengths are the same. Given that heterogeneity in variants should be one of the advantages of ctDNAmer, it seems likely that a profile of different k-mer lengths could better characterize a patient.

We expect the relatively long k-mers (>21 bp) used for ctDNA detection to capture the same variants to a large extent, although slight variations are expected. This expectation is strengthened by the correlation values between the TF estimates across k-mer lengths (Supplementary Table 1).

An ensemble approach applying a set of k-mer lengths may improve ctDNA detection. However, whether applying a set of different k-mer lengths, or rather combining the k-mer-based approach with other WGS features such as fragmentomics information, could provide the highest ctDNA detection sensitivity, warrants further analysis. We find the exploration of ensemble approaches out of scope for the current proof-of-concept study, but agree that this could improve the detection sensitivity considerably. We now mention this in the discussion section of the manuscript.

6. I believe the authors claim an improvement in computational efficiency since ctDNAmer does not rely on alignment to a reference. It would be good to provide a runtime and memory efficiency comparison to characterize the advantages and disadvantages of such a k-mer based approach.

We have noted the computational efficiency of the k-mer-based methods in the manuscript, and the efficiency of k-mer-based analysis has also been shown by previous studies [2, 3].The running time of the tool, however, depends on the k-mer length and the resources available for parallel computation. The memory and time resources needed for running ctDNAmer and clonalSNV analysis are shown in Snakemake rules of the two workflows. These, however, are upper bounds, and the actual resources used are therefore lower.

As both pipelines consist of various steps, we have not done a one-to-one comparison of memory and time usage. However, we have highlighted the average running time and memory consumption of the main ctDNAmer analysis steps in Supplementary Table 2.

7. In Figure 2f, it seems that ctDNAmer actually does differentiate across the samples characterized as negative, whereas clonal SNVs approach does not. This would seem to be a disadvantage of ctDNAmer. Can the authors add a discussion reconciling this result with the higher AUC for ctDNAmer?

Indeed, there is a larger range of ctDNA-negative samples' TF estimates seen for ctDNAmer compared with the clonal SNVs approach. While this is a disadvantage of ctDNAmer considering the estimated TF values, the AUC of the ctDNAmer tool is still improved compared to the clonal SNVs. This indicates that the clonal SNVs approach has more similar estimates, but also less power to differentiate between positive and negative samples, as reflected by the AUC. We have also added a sentence to the results section to transparently interpret the results in Figure 2F and inform the reader of this trend.

8. The authors could consider discussing the role of minimizers in this application.

We agree that the application of minimizers is a promising idea to explore for the ctDNAmer workflow. Minimizers could be used throughout the workflow instead of k-mers, starting from the analysis of primary tumor and matched germline raw sequencing reads. Alternatively, minimizers could be applied instead of the UT k-mer set filtering step, limiting the set size by counting minimizers from the candidate UT set.

Application of minimizers might simplify or completely remove the UT set filtering step and provide an improvement to the proposed method both in terms of accuracy and computational considerations. However, testing this idea would require larger considerations towards method design and implementation and we find that this is out of scope for the current proof-of-the-concept manuscript.

Nevertheless, we have added a note about the potential application of minimizers to the manuscript discussion.

9. One of the key advantages to ctDNAmer is the ability to identify different variant types and thus improve detection sensitivity. Could the authors compare the variants identified by ctDNAmer with the alignment + variant calling approaches?

Indeed, as ctDNAmer captures all sequences unique to the tumor genome we expect the UT k-mers capture variants of different types. In the revised manuscript, we have aligned the UT k-mers to the human reference genome and analysed the mapping characteristics as well as the alignments overlap with variants called by two state-of-the-practice variant calling tools: Mutect2 for SNVs and indels, and Delly for structural variants. The results can be seen in results section 3 and Supplementary Figure 5, and show that UT k-mers do overlap with variants of different types.

10. Could the k-mer length analysis be extended to evaluate whether there is a representative sample of SNVs, complex variants or structural variants at different lengths?

We have added mapping analysis for k-mer lengths of 51 and 41 and shown the overlap between the variants identified in Supplementary Figure 5. Mapped k-mers of length 41 capture 58.9% of the Mutect2 variants captured with mapped 51-mers and 86.5% of the Delly variants captured with mapped 51-mers (Supplementary Figure 5D). The analysis could not be carried out with 31-mers or shorter k-mer lengths, as only 3.77% of UT 31-mers aligned to the reference genome.

11. Could the authors provide a more intuitive explanation for why approaches leveraging additional reference data do not produce better tumor fraction estimates than ctDNAmer?

Reference data in this setting does not provide an “additional” data source, but a basis for sequence comparison of the patient data. Tumor-specific variants are identified based on the sample and reference data comparison, both when performing variant calling from the primary tumor and when identifying tumor-specific variants from cfDNA. Therefore, the reference genome is not an additional data source but the baseline against which differences are detected. The main reason why a reference-free approach might be preferred and provide more accurate results is the fact that the reference genome is not a perfect representation of the human genome. This is especially true when considering ancestry and rare germline variants that are often not well represented in the reference genome. The reference genome will also lack the patient-specific somatic variants present in the healthy cells of the patient. Therefore, the most complete sequence for sample comparison in the setting where the goal is to identify tumor-specific variants, is the healthy genome of the given individual. In the ctDNAmer workflow, the matched germline sample, used for candidate UT k-mers identification, can be viewed as the reference data and provides a better representation of the healthy genome of the individual than the human reference genome hg38 or T2T. We have taken into account the view of the matched germline as the reference data in the revised version of the manuscript, and now refer to the method as alignment-free, rather than reference-free.

12. A set of simulations would help the reader understand the role of modeling the two sources of noise.

We agree that synthetic admixture samples with different levels of contribution from germline and noise k-mers could provide an improved and more intuitive understanding of how the three-component mixture model works and how performance changes across samples with different composition. We have carried out a limit-of-detection analysis with synthetic admixture samples with different TFs (see Supplementary Figure 6), but find that the additional simulations might be out-of-scope for the current paper.

Instead, we have included a Supplementary Figure 13 which shows posterior samples' distributions (trace plots) of the key parameters for the preoperative cfDNA sample of one patient and k-mer assignments to the three components, and component weights for all cfDNA samples available for the same patient.

13. I have checked the github and read some of the additional links, but did not try running the method. I would suggest to the authors to upload a small test dataset to make it easy for the user to try running. Also, some of the links on https://github.com/BesenbacherLab/ctDNAmer/tree/main/config seem to be broken.

Links in the config and README.md files have been fixed, and we have also added a small synthetic test data set containing the two donor samples for empirical noise estimation, primary tumor, matched germline, and one cfDNA sample.

14. (minor) cfDNA definition

We assume that this review comment is referring to the fact that a definition of cfDNA is missing from the introduction. We have added the definition of cfDNA to the first subsection of the introduction.

Reviewer #3

In this expansive manuscript, the authors propose a novel approach to analysing circulating tumour DNA and apply their approach to data collected from colorectal cancer patients follow surgical resection of the primary tumour. This manuscript is a nice use-case and illustrates the potential benefits of the proposed approach. I have a few comments on the current manuscript.

We highly appreciate the reviewers' comments regarding the potential benefits of the proposed alignment-free approach and its demonstrated use case. We have addressed specific comments by revising the manuscript text and organization of the sections.

1. It was not clear to me how the authors decided on a value of k (other than requiring it to be an odd number). This seems to be an important aspect of the approach and it wasn't clear how this value was chosen, nor how the results depend on this parameter.

The k-mer length can be viewed as a hyperparameter of the ctDNAmer approach. The default value in the implemented workflow, as well as the value used in the main results section of the manuscript for the

colorectal cancer cohort, was set to 51. But the results shown in the Supplementary Table 1 also show that the performance is not very sensitive to the specific choice of k. It is usual to use only odd values of k in k-mer analyses, since k-mers that are their own reverse complement can be problematic to handle. Restricting k to be odd ensures that no k-mer will be its own reverse complement. Furthermore, the k-mers need to be long enough so that a sufficient number of UT k-mers are identified for downstream analysis. Our experiments show that analysis is feasible with k-mers of length 21 (see Supplementary Figure 7), while k-mers of length 11 result in empty UT sets (results not shown).

The TF estimation and ctDNA detection results dependence on the k-mer length parameter are shown in Supplementary Figure 7. While the results are consistent across k-mer lengths for the FRFR cohort, shorter k-mer lengths improve performance for the FFPE cohort. This illustrates that the k-mer length can be viewed as a hyperparameter. We have made a note in the discussion that the users are encouraged to test for an optimal k-mer length for their sample cohort before applying ctDNAmer.

2. The authors state that they remove " k-mers with low or high counts"; what is the threshold for removal at the low and high end? How does this decision to remove low counts influence the ability of this approach to identify de novo mutations within the tumour (as might be the case in the development of adaptive resistance to targeted therapies; I appreciate the authors are considering surgically resected tumours here, but my impression is that ctDNA could be a useful biomarker to identify the selection of resistant mutants during small molecule treatment and I wonder if this approach would be applicable in the non-surgical case).

The tumor count cutoffs applied to filter the UT sets are patient-specific. These cutoffs are found by gradually narrowing the range of included counts toward the value expected to have the highest signal-to-noise ratio (mean count of all tumor k-mers). We have included Supplementary Table 3 in the revised version, which shows the final applied count cutoffs, along with the mean tumor count and final size of the UT set for each patient in the colorectal cancer cohort.

Filtering low-count k-mers will remove UT k-mers capturing subclonal low-frequency tumor variants from the analysis, which is acceptable here, as higher frequency variants are more likely to be observed in the cfDNA samples in the low tumor burden settings. Indeed, the k-mer-based approach in principle could also be applied in a tumor-agnostic setting where the focus would be on k-mers unique to the cfDNA sample compared to the healthy genome. In this setting, k-mers could be used to identify potential resistance variants. This analysis should consider all unique k-mers without any count-based filtering, as k-mers capturing rare resistance mutations are expected to be observed one or few times.

3. On page 10, the authors give the p value from the Mann-Whitney test, which may be skewed by the large number of samples. I would appreciate if the authors reported not just the mean, but also a measure of the heterogeneity of the TF fraction here. Further, I'm not sure how to interpret the threshold of 6x10^{-4} for recurrence- do they authors expect this threshold to extent to other settings? I would appreciate more details on how this may be extended outside of the current retrospective study.

In addition to the group means, we have added measures of heterogeneity (standard deviation, median and interquartile range) for each group, and we supplement the Mann-Whitney U test with an effect-size and confidence interval. Specifically, the Hodges-Lehmann median difference and Wilcoxon CI between

groups is 0.00065 (95% CI: 0.00038, 0.00085), and Cliff's delta is 0.58492 (95% CI: 0.49455, 0.66278), indicating a large effect.

The ctDNA detection threshold is not expected to apply to other settings, except in specific cases where the biological and technical details of the cohort match those of the analysed colorectal cancer cohort. To extend the application of ctDNAmer beyond the retrospective study, either a global, fixed detection cutoff needs to be established first, or the user can set their detection cutoff based on the cohort and use case. Some settings may require enforcing a minimum sensitivity or specificity threshold. We have added sensitivity values at a fixed specificity threshold of 95% to the results section 3 and show the corresponding confusion matrices of the detection results in Supplementary Figure 3. Users can find the fitting cutoff for their data in the same manner by maximizing Youden's J or fixing the minimum specificity/sensitivity threshold. If a global detection cutoff were to be established, additional testing is first needed to understand how detection performance changes depending on the cancer type, sample coverage, and tumor purity, and then, for example, z-scores could be applied for detection, following the example of MRD-EDGE [4].

4. I wonder if the 4th section of the results could be moved to the supplemental information, as this seems to be a validation/comparison of the proposed approach against existing methods.

While we agree that the results in the 4th (and also in the 5th) section are supplemental to the central topic of the manuscript, which introduces the ctDNAmer method, we find the comparison with orthogonal methods in this setting is indispensable to prove that TF estimation and ctDNA detection with the proposed novel method are valid and accurate. Therefore, we have decided to keep the 4th and 5th sections in the main text for the presented revised version of the manuscript.

5. I found the filtering section of the methods to be difficult to follow. I think this is a crucial part of the manuscript and approach, and wonder if it would be easier to follow with a schematic/figure demonstrating how the samples are filtered to identify the UT k-mers.

The process of UT sets filtering is explained in methods section 3.1 and visualized in Supplementary Figure 10. The Supplementary Figure 10 already should outline the filtering schematically. Nevertheless, we have also tried to slightly adjust the section 3.1 further in the revised version of the manuscript to improve readability, while keeping the relevant details.

6. The authors match the maximal variance of the probabilistic model to the what was observed in the data. As the data may not fully account fo the potential variance in cfDNA and ctDNA (due to sampling difficulties etc.), I was wondering how crucial this assumption is and, more importantly, if this assumption may be imposing additional homogeneity on the data. Further, I would appreciate seeing the weights of the mixture distribution (or even a few representative examples) to see how distinct the ctDNA/Germline/noise distributions are.

Indeed, we match the variance of k-mers assigned to the germline component with the variance of matched germline k-mers observed in the same cfDNA sample and the tumor component k-mers variance is limited with a hyperparameter. We agree with the reviewer that the model is, in this case, limited to what we observe in the data, even though the true variance might be larger. Nevertheless, we

have currently kept this solution that the probabilistic model uses an Empirical Bayes approach, as without these bounds, the model can run into convergence issues. Nevertheless, these limits and the convergence issues they address affect only a small number of samples, where very little signal seems to be present in the data.

We have added a Supplementary Figure 13 that shows which shows one preoperative cfDNA sample k-mer assignments to the three components, component weights, and trace plots of the key parameters, and for the same patient, k-mer assignments to the three components and component weights are also shown for all postoperative samples.

References

1. Frydendahl, A., Nors, J., Rasmussen, M. H., Henriksen, T. V., Nesic, M., Reinert, T., Afterman, D., Lauterman, T., Kuzman, M., Gonzalez, S., Glavas, D., Smadback, J., Maloney, D., Levatic, J., Yahalom, M., Ptashkin, R., Tavassoly, I., Donenhirsh, Z., White, E., . . . Andersen, C. L. (2024). Detection of circulating tumor DNA by tumor-informed whole-genome sequencing enables prediction of recurrence in stage III colorectal cancer patients. European Journal of Cancer, 211, 114314. https://doi.org/10.1016/j.ejca.2024.114314
2. Wang, Y., Xue, H., Pourcel, C. et al. 2-kupl: mapping-free variant detection from DNA-seq data of matched samples. BMC Bioinformatics 22, 304 (2021). https://doi.org/10.1186/s12859-021-04185-6
3. Chen, S., Huang, T., Wen, T. et al. MutScan: fast detection and visualization of target mutations by scanning FASTQ data. BMC Bioinformatics 19, 16 (2018). https://doi.org/10.1186/s12859-018-2024-6
4. Widman, A.J., Shah, M., Frydendahl, A. *et al.* Ultrasensitive plasma-based monitoring of tumor burden using machine-learning-guided signal enrichment. *Nat Med* 30, 1655–1666 (2024). https://doi.org/10.1038/s41591-024-03040-4
