# Supplementary material for: An alignment-free strategy for circulating tumor DNA detection and tumor fraction estimation from whole-genome sequencing data
Source: PLoS Comput Biol. 2026 Aug 10;22(8):e1013356. doi: 10.1371/journal.pcbi.1013356 (PMC13485129; doi:10.1371/journal.pcbi.1013356)
Supplement: Supplementary file 3 [file pcbi.1013356.s022.pdf]

Point-by-point responses to the reviewers

Below, reviewers' comments and questions are shown in black, and our responses in blue.

Reviewer #2

I could not find where the authors mention temporal tumor heterogeneity as a limitation. Given that the sample-level sensitivity is 54%, subclonal evolution is a plausible contributor that should be acknowledged in the Discussion (again, apologies if it's there and I could not find it).

Answer: We thank the reviewer for pointing this out. We agree that subclonal evolution represents an important limitation of tumor-informed ctDNA detection approaches that rely on variants identified from the primary tumor sample. We have now added a note to the 5th paragraph of the discussion section acknowledging that changes in tumor clonal composition during disease progression could reduce the representation of some tumor-specific k-mers in longitudinal cfDNA samples and thereby affect detection sensitivity.

The authors did not add a sensitivity analysis to show what happens when the window around the mean tumor count changes.

Answer: We thank the reviewer for bringing this to our attention. We have now added a sensitivity analysis evaluating the effect of varying the UT k-mer count filtering window by expanding the empirically selected lower and upper tumor count cutoffs by ±2 and +4 counts (Supplementary Table 1) and referenced this sensitivity analysis in the results section. Expanding the filtering window increased the size of the resulting UT sets but generally reduced overall detection performance, particularly in terms of AUC and specificity, although a modest increase in sensitivity was observed in the FRFR cohort. These results support the use of the empirically selected count cutoffs as a reasonable balance between sensitivity and specificity.

The biological validation with Mutect2/Delly does not apply for the FFPE cohort where k=21 is optimal. The authors should explicitly state this as a limitation.

Answer: We agree that the biological validation analysis based on overlap with Mutect2 and DELLY variant calls could not be performed for the FFPE cohort at k=21 because short 21-mers aligned ambiguously to the reference genome. We have now explicitly acknowledged this limitation in the manuscript at the end of the 6th result section.

The subsequent discussion of unique advantages present in WGS-based ctDNA detection could still be clearer. The authors could add a more direct discussion of how alignment-free detection might complement existing alignment-based approaches.

Answer: We thank the reviewer for this suggestion. We agree that the potential complementary role of alignment-free ctDNA detection alongside existing alignment-based approaches could be explained more clearly. We have now added a discussion paragraph emphasizing that ctDNAmer is not intended to

replace alignment-based workflows, but rather may provide orthogonal sequence-level information that could be integrated with alignment-based features.

If computational efficiency will be mentioned in the main paper, the authors should at least highlight one quantitative comparison in the main paper. I do see that the authors added a reference to the supplementary table in the discussion, but it is easier on the reader to add example numbers as a frame of reference. Indeed, the authors include Supplementary Table 2 without any units, so that the mean running time sum of ~20 is essentially meaningless. The point that a k-mer based approach is faster is intuitive but is also not demonstrated in this table.

Answer: We thank the reviewer for pointing this out. The previous version of Supplementary Table 2 lacked units due to human error and therefore did not provide sufficient quantitative context for the computational requirements of ctDNAmer. We have now added the corresponding units (hours and GB RAM) to Supplementary Table 2 and included representative runtime values directly in the main Discussion text to provide a clearer frame of reference for the reader. We also adjusted the wording in the manuscript to avoid claiming computational efficiency, as a direct comparison of computational resource usage across different methods is out of scope for the current study.

I would still like to see the authors add a few sentences to Methods (apologies if I could not find it) clearly stating all the inputs necessary to run the method, and what outputs are produced.

Answer: We thank the reviewer for this suggestion. We have now added a section to the beginning of the Methods section (“Input and output data”) describing the required sequencing inputs, main configurable workflow parameters, and the main outputs produced by ctDNAmer. Input data is further exemplified by the test data set added to GitHub during the first review round.

The original comment about the simulation was to understand what happens when the germline noise vs. technical noise contributions vary. Supplementary Figure 13 does not describe noise composition – if out of scope, perhaps it would be better to add to limitations.

Answer: We thank the reviewer for bringing this to our attention. We agree that Supplementary Figure 13 does not directly address how the probabilistic model behaves under varying contributions of germline and technical noise. As a systematic simulation analysis of noise composition changes is out of scope for the current study, we have now explicitly acknowledged this as a limitation in the second to last paragraph of the Discussion section.

Reviewer #3

The authors have satisfactorily addressed all my concerns. I appreciate the authors adding additional information and analyses to explore the role of the method's "hyper parameters" on the results. I think it might be worthwhile to explicitly list the assumptions underlying these hyperparameters, potentially in the SI, along with a clear explanation of which assumptions can potentially be relaxed, as in the response to my 2nd comment on the initial submission.

Answer: We thank the reviewer for the positive assessment of the revised manuscript and the suggestion for summarizing the assumptions explicitly. We have added a supplementary note (“Supplementary Note 1. Rationale and potential relaxation of the ctDNAmer workflow assumptions.”) describing the rationale

behind the main hyperparameters and the underlying assumptions, together with examples of how these assumptions could potentially be relaxed in alternative applications.
