# Supplementary material for: An alignment-free strategy for circulating tumor DNA detection and tumor fraction estimation from whole-genome sequencing data
Source: PLoS Comput Biol. 2026 Aug 10;22(8):e1013356. doi: 10.1371/journal.pcbi.1013356 (PMC13485129; doi:10.1371/journal.pcbi.1013356)
Supplement: Supplementary file 4 [file pcbi.1013356.s001.docx]

# S1 Text

***Supplementary Note 1. Rationale and potential relaxation of the ctDNAmer workflow assumptions.***

The ctDNAmer workflow includes user-defined hyperparameters that reflect assumptions about the expected properties of unique tumor k-mers in WGS data. These assumptions were selected to improve the signal-to-noise ratio in low-tumor-burden settings but could potentially be modified depending on the intended application of the method.

The choice of k-mer length represents a tradeoff between sequence uniqueness and robustness to sequencing artifacts. Longer k-mers are more likely to uniquely represent tumor-derived sequence changes and less likely to align ambiguously to the reference genome, whereas shorter k-mers are more abundant and may increase sensitivity to degraded DNA fragments, but also increase the probability of ambiguous mapping and contribution of background noise. The optimal k-mer length may therefore depend on sequencing quality, read length, sample preservation method, and tumor mutational characteristics. In this study, longer k-mers performed better in the FRFR cohort, whereas shorter k-mers improved performance in the FFPE cohort, likely due to the higher degree of DNA fragmentation in FFPE-derived material.

The GC-content filtering thresholds assume that k-mers with extreme GC content are more susceptible to sequencing and amplification biases and, therefore, less likely to provide stable tumor fraction estimates. Relaxing these thresholds could increase the number of retained unique tumor k-mers and potentially improve sensitivity in some cases, but may also increase technical variability and noise. Future implementations could replace fixed GC-content thresholds with sequencing platform-specific bias correction approaches.

The UT k-mer count filtering assumes that low-count k-mers are more likely to represent sequencing artifacts or low-frequency subclonal variants that are unlikely to be reliably observed in low tumor burden cfDNA samples. Similarly, high-count k-mers are assumed to originate more frequently from repetitive or non-unique genomic regions. Relaxing the lower count cutoff could improve sensitivity to rare tumor subclones or emerging resistance-associated variants, particularly in tumor-agnostic or longitudinal monitoring settings, but would likely increase susceptibility to technical noise. Relaxing the upper count cutoff could increase the inclusion of repetitive sequence-derived k-mers and reduce specificity. In the current implementation, patient-specific tumor count cutoffs were applied to filter the UT sets. These cutoffs were found by gradually narrowing the range of included counts toward the value expected to have the highest signal-to-noise ratio (mean count of all tumor k-mers).
