# Supplementary material for: An alignment-free strategy for circulating tumor DNA detection and tumor fraction estimation from whole-genome sequencing data
Source: PLoS Comput Biol. 2026 Aug 10;22(8):e1013356. doi: 10.1371/journal.pcbi.1013356 (PMC13485129; doi:10.1371/journal.pcbi.1013356)
Supplement: Peer review history

# Peer review: Decision Letter 0

**DOI:** 10.1371/journal.pcbi.1013356.r001

**Authors:** Shaun Mahony, Philipp Altrock

29 Sep 2025

A reference-free strategy for circulating tumor DNA detection from whole-genome sequencing data

PLOS Computational Biology

Dear Dr. Besenbacher,

Thank you for submitting your manuscript to PLOS Computational Biology. After careful consideration, we feel that it has merit but does not fully meet PLOS Computational Biology's publication criteria as it currently stands. Therefore, we invite you to submit a revised version of the manuscript that addresses the points raised during the review process.

Please submit your revised manuscript within 60 days Nov 29 2025 11:59PM. If you will need more time than this to complete your revisions, please reply to this message or contact the journal office at ploscompbiol@plos.org. When you're ready to submit your revision, log on to https://www.editorialmanager.com/pcompbiol/ and select the 'Submissions Needing Revision' folder to locate your manuscript file.

Please include the following items when submitting your revised manuscript:

A rebuttal letter that responds to each point raised by the editor and reviewer(s). You should upload this letter as a separate file labeled 'Response to Reviewers'. This file does not need to include responses to formatting updates and technical items listed in the 'Journal Requirements' section below.

* A marked-up copy of your manuscript that highlights changes made to the original version. You should upload this as a separate file labeled 'Revised Manuscript with Track Changes'.

* An unmarked version of your revised paper without tracked changes. You should upload this as a separate file labeled 'Manuscript'.

If you would like to make changes to your financial disclosure, competing interests statement, or data availability statement, please make these updates within the submission form at the time of resubmission. Guidelines for resubmitting your figure files are available below the reviewer comments at the end of this letter

We look forward to receiving your revised manuscript.

Kind regards,

Philipp Martin Altrock, Ph.D.

Academic Editor

PLOS Computational Biology

Shaun Mahony

Section Editor

PLOS Computational Biology

**Journal Requirements:**

1) We ask that a manuscript source file is provided at Revision. Please upload your manuscript file as a .doc, .docx, .rtf or .tex. If you are providing a .tex file, please upload it under the item type u2018LaTeX Source Fileu2019 and leave your .pdf version as the item type u2018Manuscriptu2019.

2) Please provide an Author Summary. This should appear in your manuscript between the Abstract (if applicable) and the Introduction, and should be 150-200 words long. The aim should be to make your findings accessible to a wide audience that includes both scientists and non-scientists. Sample summaries can be found on our website under Submission Guidelines:

https://journals.plos.org/ploscompbiol/s/submission-guidelines#loc-parts-of-a-submission

3) Thank you for including an Ethics Statement for your study. Please include:

i) A statement that formal consent was obtained (must state whether verbal/written) OR the reason consent was not obtained (e.g. anonymity). NOTE: If child participants, the statement must declare that formal consent was obtained from the parent/guardian.].

4) Please upload all main figures as separate Figure files in .tif or .eps format. For more information about how to convert and format your figure files please see our guidelines:

https://journals.plos.org/ploscompbiol/s/figures

5) We notice that your supplementary Figures are included in the manuscript file. Please remove them and upload them with the file type 'Supporting Information'. Please ensure that each Supporting Information file has a legend listed in the manuscript after the references list.

6) Some material included in your submission may be copyrighted. According to PLOSu2019s copyright policy, authors who use figures or other material (e.g., graphics, clipart, maps) from another author or copyright holder must demonstrate or obtain permission to publish this material under the Creative Commons Attribution 4.0 International (CC BY 4.0) License used by PLOS journals. Please closely review the details of PLOSu2019s copyright requirements here: PLOS Licenses and Copyright. If you need to request permissions from a copyright holder, you may use PLOS's Copyright Content Permission form.

Please respond directly to this email and provide any known details concerning your material's license terms and permissions required for reuse, even if you have not yet obtained copyright permissions or are unsure of your material's copyright compatibility. Once you have responded and addressed all other outstanding technical requirements, you may resubmit your manuscript within Editorial Manager.

Potential Copyright Issues:

i) Figures 1A, and 1C. Please confirm whether you drew the images / clip-art within the figure panels by hand. If you did not draw the images, please provide (a) a link to the source of the images or icons and their license / terms of use; or (b) written permission from the copyright holder to publish the images or icons under our CC BY 4.0 license. Alternatively, you may replace the images with open source alternatives. See these open source resources you may use to replace images / clip-art:

- https://commons.wikimedia.org

- https://openclipart.org/.

7) Please amend your detailed Financial Disclosure statement. This is published with the article. It must therefore be completed in full sentences and contain the exact wording you wish to be published.

1) If the funders had no role in your study, please state: "The funders had no role in study design, data collection and analysis, decision to publish, or preparation of the manuscript."

8) Thank you for stating "The authors declare the following financial interests/personal relationships, which may be considered as potential competing interests. CLA reports sponsored research agreements with Natera, Bio-Rad Laboratories, AccuraGen Inc., and Labcorp." Please modify your 'Competing Interests' statement in the online submission form and declare all competing interests beginning with the statement "I have read the journal's policy and the authors of this manuscript have the following competing interests:"

Note: If the reviewer comments include a recommendation to cite specific previously published works, please review and evaluate these publications to determine whether they are relevant and should be cited. There is no requirement to cite these works unless the editor has indicated otherwise.

**Reviewers' comments:**

Reviewer's Responses to Questions

**Comments to the Authors:**

**Please note that one review is uploaded as an attachment.**

Reviewer #1: In this manuscript, Oroperv and colleagues propose a reference free, “tumor informed” cfDNA approach based on k mer analysis across three matched samples per patient, tumor, germline, and plasma cfDNA; each sequenced at ~30× whole genome depth (on the order of hundreds of millions of fragments per sample). The model is then applied to a cohort of stage III colorectal cancer patients (n=90) with three year follow up.

From a computational/bioinformatics standpoint, the technique is novel and could have utility beyond the specific use case studied. However, as presented, the performance and clinical applicability are unclear and underwhelming. The strategy effectively requires ~90× WGS per patient (3×30×), yet the detection limit appears to lie between ~0.1% and 1% (Figure 2F), which substantially limits clinical utility and is not competitive in the MRD setting. By comparison, simple targeted capture methods focusing on recurrent SNVs can reach limits around ~0.01% at roughly an order of magnitude lower cost than ~90× WGS. Moreover, for standard ~30× WGS, alignment is performed in routine workflows; thus, the “reference free” paradigm does not realistically prevent mapping. That said, I would be interested to see whether k mer–based signals provide orthogonal information that synergizes with post alignment features (e.g., variants, copy number, indels, fragment length).

I encourage the authors to consider reframing the contribution toward contexts such as novel sequence identification or motif discovery, rather than MRD detection or circulating tumor fraction estimation. Hence, I recommend major revision.

Major Comments

1. The k mer analysis is highly computationally intensive, requiring analysis of billions of reads. Without alignment, it is also difficult to confirm the biological validity of findings—e.g., whether observed signals correspond to mutations relevant to the cancer type of interest.

2. Please comment on the impact of PCR induced duplications on the k mer counts and downstream results. How are duplicates detected/handled, and how sensitive are results to duplication rates?

3. The selected k values (31, 41, 51, 61) appear primarily empirical. While k=51 performed best in FRFR samples and k=31 in FFPE samples, the rationale for limiting the search to this range is not fully clear. Why were multi k strategies not explored? Since the optimal k seems to differ by sample type, please discuss whether broader sensitivity analyses or adaptive k mer selection might improve robustness across sequencing contexts.

4. On page 21, the manuscript suggests that smaller k mers yield better results, which contrasts with earlier choices and highlights the framework’s sensitivity to k. This raises robustness concerns that should be addressed explicitly.

5. Consider mapping reads carrying “germline” k mers back to the reference and testing for enrichment of known population SNPs (e.g., from gnomAD) within a statistical framework to substantiate the interpretation of these signals.

6. When reporting sensitivity and specificity, please also provide sensitivity at clinically relevant fixed specificity thresholds (e.g., 95% or 98%), which facilitates comparison to established assays.

7. The manuscript lacks an LOD analysis, which is standard for evaluating detection frameworks. This could be addressed via in silico spike in (mixture) experiments across a range of tumor fractions.

Reviewer #2: Uploaded as attachment

Reviewer #3: In this expansive manuscript, the authors propose a novel approach to analysing circulating tumour DNA and apply their approach to data collected from colorectal cancer patients follow surgical resection of the primary tumour. This manuscript is a nice use-case and illustrates the potential benefits of the proposed approach. I have a few comments on the current manuscript.

1) It was not clear to me how the authors decided on a value of k (other than requiring it to be an odd number). This seems to be an important aspect of the approach and it wasn't clear how this value was chosen, nor how the results depend on this parameter.

2) The authors state that they remove " k-mers with low or high counts"; what is the threshold for removal at the low and high end? How does this decision to remove low counts influence the ability of this approach to identify de novo mutations within the tumour (as might be the case in the development of adaptive resistance to targeted therapies; I appreciate the authors are considering surgically resected tumours here, but my impression is that ctDNA could be a useful biomarker to identify the selection of resistant mutants during small molecule treatment and I wonder if this approach would be applicable in the non-surgical case).

3) On page 10, the authors give the p value from the Mann-Whitney test, which may be skewed by the large number of samples. I would appreciate if the authors reported not just the mean, but also a measure of the heterogeneity of the TF fraction here. Further, I'm not sure how to interpret the threshold of 6x10^{-4} for recurrence- do they authors expect this threshold to extent to other settings? I would appreciate more details on how this may be extended outside of the current retrospective study.

4) I wonder if the 4th section of the results could be moved to the supplemental information, as this seems to be a validation/comparison of the proposed approach against existing methods.

5) I found the filtering section of the methods to be difficult to follow. I think this is a crucial part of the manuscript and approach, and wonder if it would be easier to follow with a schematic/figure demonstrating how the samples are filtered to identify the UT k-mers.

6) The authors match the maximal variance of the probabilistic model to the what was observed in the data. As the data may not fully account fo the potential variance in cfDNA and ctDNA (due to sampling difficulties etc.), I was wondering how crucial this assumption is and, more importantly, if this assumption may be imposing additional homogeneity on the data. Further, I would appreciate seeing the weights of the mixture distribution (or even a few representative examples) to see how distinct the ctDNA/Germline/noise distributions are.

**********

**Have the authors made all data and (if applicable) computational code underlying the findings in their manuscript fully available?**

The PLOS Data policy requires authors to make all data and code underlying the findings described in their manuscript fully available without restriction, with rare exception (please refer to the Data Availability Statement in the manuscript PDF file). The data and code should be provided as part of the manuscript or its supporting information, or deposited to a public repository. For example, in addition to summary statistics, the data points behind means, medians and variance measures should be available. If there are restrictions on publicly sharing data or code —e.g. participant privacy or use of data from a third party—those must be specified.

Reviewer #1: Yes

Reviewer #2: Yes

Reviewer #3: Yes

**********

PLOS authors have the option to publish the peer review history of their article (what does this mean?). If published, this will include your full peer review and any attached files.

If you choose “no”, your identity will remain anonymous but your review may still be made public.

**Do you want your identity to be public for this peer review?**  For information about this choice, including consent withdrawal, please see our Privacy Policy.

Reviewer #1: No

Reviewer #2: No

Reviewer #3: No

[NOTE: If reviewer comments were submitted as an attachment file, they will be attached to this email and accessible via the submission site. Please log into your account, locate the manuscript record, and check for the action link "View Attachments". If this link does not appear, there are no attachment files.]

**Figure resubmission:**

After uploading your figures to PLOS’s NAAS tool - https://ngplosjournals.pagemajik.ai/artanalysis, NAAS will process the files provided and display the results in the "Uploaded Files" section of the page as the processing is complete. If the uploaded figures meet our requirements (or NAAS is able to fix the files to meet our requirements), the figure will be marked as "fixed" above. If NAAS is unable to fix the files, a red "failed" label will appear above. When NAAS has confirmed that the figure files meet our requirements, please download the file via the download option, and include these NAAS processed figure files when submitting your revised manuscript.

**Reproducibility:**

?>

Attachment Submitted filename: ctDNAmer review plos_compbio 09.21.2025.pdf

# Author comment: Author response to Decision Letter 1

**DOI:** 10.1371/journal.pcbi.1013356.r002

6 Feb 2026

Attachment Submitted filename: ctDNAmer rebuttal letter.pdf

# Peer review: Decision Letter 1

**DOI:** 10.1371/journal.pcbi.1013356.r003

**Authors:** Shaun Mahony, Philipp Altrock

30 Mar 2026

An alignment-free strategy for circulating tumor DNA detection and tumor fraction estimation from whole-genome sequencing data

PLOS Computational Biology

Dear Dr. Besenbacher,

Thank you for submitting your manuscript to PLOS Computational Biology. After careful consideration, we feel that it has merit but does not fully meet PLOS Computational Biology's publication criteria as it currently stands. Therefore, we invite you to submit a revised version of the manuscript that addresses the points raised during the review process.

Please submit your revised manuscript by May 30 2026 11:59PM. If you will need more time than this to complete your revisions, please reply to this message or contact the journal office at ploscompbiol@plos.org. When you're ready to submit your revision, log on to https://www.editorialmanager.com/pcompbiol/ and select the 'Submissions Needing Revision' folder to locate your manuscript file.

Please include the following items when submitting your revised manuscript:

* A letter that responds to each point raised by the editor and reviewer(s). You should upload this letter as a separate file labeled 'Response to Reviewers'. This file does not need to include responses to formatting updates and technical items listed in the 'Journal Requirements' section below.

* A marked-up copy of your manuscript that highlights changes made to the original version. You should upload this as a separate file labeled 'Revised Manuscript with Track Changes'.

* An unmarked version of your revised paper without tracked changes. You should upload this as a separate file labeled 'Manuscript'.

If you would like to make changes to your financial disclosure, competing interests statement, or data availability statement, please make these updates within the submission form at the time of resubmission. Guidelines for resubmitting your figure files are available below the reviewer comments at the end of this letter

We look forward to receiving your revised manuscript.

Kind regards,

Philipp Martin Altrock, Ph.D.

Academic Editor

PLOS Computational Biology

Shaun Mahony

Section Editor

PLOS Computational Biology

**Reviewers' comments:**

Reviewer's Responses to Questions

**Comments to the Authors:**

**Please note here if the review is uploaded as an attachment.**

Reviewer #2: I find that the authors have addressed some of my concerns, and that the manuscript is improved with the additional analyses. However, I have a few concerns remaining.

Major remaining concerns:

I could not find where the authors mention temporal tumor heterogeneity as a limitation. Given that the sample-level sensitivity is 54%, subclonal evolution is a plausible contributor that should be acknowledged in the Discussion (again, apologies if it’s there and I could not find it).

The authors did not add a sensitivity analysis to show what happens when the window around the mean tumor count changes.

The biological validation with Mutect2/Delly does not apply for the FFPE cohort where k=21 is optimal. The authors should explicitly state this as a limitation.

The subsequent discussion of unique advantages present in WGS-based ctDNA detection could still be clearer. The authors could add a more direct discussion of how alignment-free detection might complement existing alignment-based approaches.

If computational efficiency will be mentioned in the main paper, the authors should at least highlight one quantitative comparison in the main paper. I do see that the authors added a reference to the supplementary table in the discussion, but it is easier on the reader to add example numbers as a frame of reference. Indeed, the authors include Supplementary Table 2 without any units, so that the mean running time sum of ~20 is essentially meaningless. The point that a k-mer based approach is faster is intuitive but is also not demonstrated in this table.

Minor points:

I would still like to see the authors add a few sentences to Methods (apologies if I could not find it) clearly stating all the inputs necessary to run the method, and what outputs are produced.

The original comment about the simulation was to understand what happens when the germline noise vs. technical noise contributions vary. Supplementary Figure 13 does not describe noise composition – if out of scope, perhaps it would be better to add to limitations.

Reviewer #3: The authors have satisfactorily addressed all my concerns. I appreciate the authors adding additional information and analyses to explore the role of the method's "hyper parameters" on the results. I think it might be worthwhile to explicitly list the assumptions underlying these hyperparameters, potentially in the SI, along with a clear explanation of which assumptions can potentially be relaxed, as in the response to my 2nd comment on the initial submission.

**********

**Have the authors made all data and (if applicable) computational code underlying the findings in their manuscript fully available?**

The PLOS Data policy requires authors to make all data and code underlying the findings described in their manuscript fully available without restriction, with rare exception (please refer to the Data Availability Statement in the manuscript PDF file). The data and code should be provided as part of the manuscript or its supporting information, or deposited to a public repository. For example, in addition to summary statistics, the data points behind means, medians and variance measures should be available. If there are restrictions on publicly sharing data or code —e.g. participant privacy or use of data from a third party—those must be specified.

Reviewer #2: Yes

Reviewer #3: Yes

**********

PLOS authors have the option to publish the peer review history of their article (what does this mean?). If published, this will include your full peer review and any attached files.

If you choose “no”, your identity will remain anonymous but your review may still be made public.

**Do you want your identity to be public for this peer review?**  For information about this choice, including consent withdrawal, please see our Privacy Policy.

Reviewer #2: No

Reviewer #3: No

[NOTE: If reviewer comments were submitted as an attachment file, they will be attached to this email and accessible via the submission site. Please log into your account, locate the manuscript record, and check for the action link "View Attachments". If this link does not appear, there are no attachment files.]

**Figure resubmission:**

**Reproducibility:**

?>

# Author comment: Author response to Decision Letter 2

**DOI:** 10.1371/journal.pcbi.1013356.r004

13 Jun 2026

Attachment Submitted filename: Response_to_Reviewers_revision_2.pdf

# Editor report: Decision Letter 2

**DOI:** 10.1371/journal.pcbi.1013356.r005

**Authors:** Shaun Mahony, Philipp Altrock

9 Jul 2026

Dear Dr. Besenbacher,

We are pleased to inform you that your manuscript 'An alignment-free strategy for circulating tumor DNA detection and tumor fraction estimation from whole-genome sequencing data' has been provisionally accepted for publication in PLOS Computational Biology.

Before your manuscript can be formally accepted you will need to complete some formatting changes, which you will receive in a follow up email. A member of our team will be in touch with a set of requests.

Please note that your manuscript will not be scheduled for publication until you have made the required changes, so a swift response is appreciated.

IMPORTANT: The editorial review process is now complete. PLOS will only permit corrections to spelling, formatting or significant scientific errors from this point onwards. Requests for major changes, or any which affect the scientific understanding of your work, will cause delays to the publication date of your manuscript.

Should you, your institution's press office or the journal office choose to press release your paper, you will automatically be opted out of early publication. We ask that you notify us now if you or your institution is planning to press release the article. All press must be co-ordinated with PLOS.

Thank you again for supporting Open Access publishing; we are looking forward to publishing your work in PLOS Computational Biology.

Best regards,

Philipp Martin Altrock, Ph.D.

Academic Editor

PLOS Computational Biology

Shaun Mahony

Section Editor

PLOS Computational Biology

***********************************************************

# Editor report: Acceptance letter

**DOI:** 10.1371/journal.pcbi.1013356.r006

**Authors:** Shaun Mahony, Philipp Altrock

PCOMPBIOL-D-25-01343R2

An alignment-free strategy for circulating tumor DNA detection and tumor fraction estimation from whole-genome sequencing data

Dear Dr Besenbacher,

I am pleased to inform you that your manuscript has been formally accepted for publication in PLOS Computational Biology. Your manuscript is now with our production department and you will be notified of the publication date in due course.

The corresponding author will soon be receiving a typeset proof for review, to ensure errors have not been introduced during production. Please review the PDF proof of your manuscript carefully, as this is the last chance to correct any errors. Please note that major changes, or those which affect the scientific understanding of the work, will likely cause delays to the publication date of your manuscript.

Soon after your final files are uploaded, unless you have opted out, the early version of your manuscript will be published online. The date of the early version will be your article's publication date. The final article will be published to the same URL, and all versions of the paper will be accessible to readers.

For Research, Software, and Methods articles, you will receive an invoice from PLOS for your publication fee after your manuscript has reached the completed accept phase. If you receive an email requesting payment before acceptance or for any other service, this may be a phishing scheme. Learn how to identify phishing emails and protect your accounts at https://explore.plos.org/phishing.

Thank you again for supporting PLOS Computational Biology and open-access publishing. We are looking forward to publishing your work!

With kind regards,

Sharmila Kamatchi

PLOS Computational Biology | Carlyle House, Carlyle Road, Cambridge CB4 3DN | United Kingdom ploscompbiol@plos.org | Phone +44 (0) 1223-442824 | ploscompbiol.org | @PLOSCompBiol
